# Enhancer of the rudimentary gene homologue (*ERH*) expression pattern in sporadic human breast cancer and normal breast tissue

**DOI:** 10.1186/1471-2407-8-145

**Published:** 2008-05-23

**Authors:** Menelaos Zafrakas, Inge Losen, Ruth Knüchel, Edgar Dahl

**Affiliations:** 1 Molecular Oncology Group, Institute of Pathology, University Hospital of the RWTH Aachen, Aachen, Germany

## Abstract

**Background:**

The human gene *ERH *(Enhancer of the Rudimentary gene Homologue) has previously been identified by *in silico *analysis of four million ESTs as a gene differentially expressed in breast cancer. The biological function of ERH protein has not been fully elucidated, however functions in cell cycle progression, pyrimidine metabolism a possible interaction with p21(Cip1/Waf1) via the Ciz1 zinc finger protein have been suggested. The aim of the present study was a systematic characterization of *ERH *expression in human breast cancer in order to evaluate possible clinical applications of this molecule.

**Methods:**

The expression pattern of *ERH *was analyzed using multiple tissue northern blots (MTN) on a panel of 16 normal human tissues and two sets of malignant/normal breast and ovarian tissue samples. *ERH *expression was further analyzed in breast cancer and normal breast tissues and in tumorigenic as well as non-tumorigenic breast cancer cell lines, using quantitative RT-PCR and non-radioisotopic *in situ *hybridization (ISH).

**Results:**

Among normal human tissues, *ERH *expression was most abundant in testis, heart, ovary, prostate, and liver. In the two MTN sets of malignant/normal breast and ovarian tissue,*ERH *was clearly more abundantly expressed in all tumours than in normal tissue samples. Quantitative RT-PCR analyses showed that *ERH *expression was significantly more abundant in tumorigenic than in non-tumorigenic breast cancer cell lines (4.5-fold; p = 0.05, two-tailed Mann-Whitney U-test); the same trend was noted in a set of 25 primary invasive breast cancers and 16 normal breast tissue samples (2.5-fold; p = 0.1). These findings were further confirmed by non-radioisotopic ISH in human breast cancer and normal breast tissue.

**Conclusion:**

*ERH *expression is clearly up-regulated in malignant as compared with benign breast cells both in primary human breast cancer and in cell models of breast cancer. Since similar results were obtained for ovarian cancer, ERH overexpression may be implicated in the initiation and/or progression of certain human malignancies. Further studies on large breast cancer tissue cohorts should determine whether ERH could function as a prognostic factor or even a drug target in the treatment of human breast cancer.

## Background

The human *ERH *(Enhancer of the Rudimentary gene Homologue) gene encodes a protein highly conserved among eukaryotes. It has been identified after comparison of human ESTs with known genes in public databases, as a gene highly homologous to the enhancer of the rudimentary gene (DROER) in *Drosophila melanogaster *[1]. The human *ERH *consists of 797 nucleotides, including an open reading frame of 312 nucleotides, encoding a protein of 104 amino acids. *ERH *has been mapped to the chromosomal band 7q34 by fluorescence *in situ *hybridization, and its expression was originally found in all normal human tissues examined [1]. Details on the purification and crystallization of the human ERH protein have been reported recently [2].

Intriguingly, the enhancer of the rudimentary gene – named *ERH *in humans, *er(h) *in all non-human species, *DROER *in Drosophila and *XERH *in Xenopus – is highly conserved among vertebrates, invertebrates, and plants with various orthologs identified, while there are no homologous sequences known within the same species [3-5]. The human and mouse coding regions are 93% identical, and the amino acid sequence of their proteins are completely identical to each other, as well as to that of the frog (*Xenopus laevis*) [3,4]. Furthermore, the human ERH protein has a 79.8% identity in amino acid sequence to that of *D. melanogaster *[1]. Similarly impressive is the conservation of hydrophobic amino acids: Of the 27 positions occupied by hydrophobic amino acids in DROER, 25 (93%) are conserved in the mosquito and human, 23 (85%) in the nematode (*C. elegans*), and 20 (74%) in the *Arapidopsis thaliana *protein [3].

Using the *in silico *method electronic Northern (eNorthern) for RNA expression profiling, we have previously identified a genetic signature containing hundreds of candidate genes differentially expressed in breast and ovarian cancer [6]. Characterization of a subset of these candidate genes, by cDNA dot blot using cancer profiling arrays, real-time RT-PCR, non radioisotopic RNA *in situ *hybridization (ISH) and immunohistochemistry has been reported elsewhere [7-11]. *ERH *was identified by this *in silico *approach among other genes, and this gave us the impetus to further study its expression in human breast cancer. In the present study, we present a systematic expression analysis of *ERH *in a panel of breast cancer cell lines and malignant and normal human breast tissue samples using Northern blot, quantitative RT-PCR and non-radioisotopic RNA ISH.

## Methods

### Tissue Specimens and RNA extraction

Formalin-fixed paraffin-embedded tissue from breast cancer and corresponding normal tissue specimens were obtained from patients treated at the Gynaecology Department of the University Hospital of Aachen, with institutional review board approval. The cohort of breast tissue specimens analyzed in this study (25 human invasive breast cancers and 16 unmatched normal breast tissues) has been described previously [9]. The histopathological data of the tumours are summarized in Table 1. For each formalin-fixed paraffin-embedded tissue specimen six 4-μm thick tissue sections were cut with a microtome (Leica, Wetzlar, Germany) and transferred to a water bath filled with DEPC-treated water. Sections were mounted on standard glass slides, dried for 1 h at 60°C, and deparaffinized and rehydrated as follows: 2 × 15 min in xylole, 2 × 15 min in 100% ethanol, and short rinses in 96%, 70%, 50% ethanol followed by emersion in distilled water. Tissue material was transferred to a microcentrifuge tube and RNA was extracted according to the Trizol protocol supplied by the manufacturer (Life Technologies, Mannheim, Germany).

**Table 1:** Baseline characteristics of primary breast carcinomas (n = 25)

**Variable**	**Categorization**	**n analyzable^a^**	**%**
***Clinico-pathologic data:***			
Age at diagnosis			
	median 63.7 years (range 35–83 years)		
	<50 years	3	12
	= 50 years	22	88
Tumour stage^b^			
	pT1	10	40
	pT2	10	40
	pT3	3	12
	pT4	2	8
Lymph node status^b^			
	pN0	12	48
	pN1	1	4
	pN2	8	32
	pN3	4	16
Histologic grade			
	G1	0	0
	G2	10	40
	G3	15	60
Histologic type			
	ductal	20	80
	lobular	3	12
	mixed ductal/lobular	1	4
	other	1	4
			
***Immunohistochemistry (IHC):***			
Estrogen receptor status			
	negative (IRS^c ^0–2)	5	20
	positive (IRS 3–12)	20	80
Progesterone receptor status			
	negative (IRS 0–2)	7	28
	positive (IRS 3–12)	18	72
HER2 IHC			
	unknown	2	8
	negative (0–1+)	19	76
	positive (2+–3+)	4	16

^a ^only female patients with primary, unilateral, invasive breast cancer were included

^b ^according to UICC: TNM Classification of Malignant Tumours. 6th edn (2002) Sobin LH, Wittekind CH (eds.) Wiley, New York.

^c ^IRS: immune-reactivity score

### Cell lines and RNA extraction

The non-tumorigenic breast cancer cell lines MCF12A and MCF10A, and five tumorigenic breast cancer cell lines (MCF7, SKBR3, T47D, ZR75-1, and BT-20) were obtained from the American Type Culture Collection (ATCC, Rockville, MD, USA) and cultured under recommended conditions. RNA from cell lines was extracted using the Trizol protocol (see above).

### RNA expression analysis by northern blot in normal and malignant human tissues

*ERH *expression was analyzed by multiple tissue northern blots (MTN) in a panel of 16 normal tissues, a set of four matched breast cancer/normal breast, and a set of four matched ovarian cancer/normal ovarian tissue samples (Clontech, Heidelberg, Germany). The following normal tissues were analyzed: heart, brain, placenta, lung, liver, skeletal muscle, kidney, pancreas, spleen, thymus, prostate, testis, ovary, small intestine, colon, and peripheral blood leukocytes. The breast cancer MTN contained four pairs of invasive ductal carcinoma and matched normal breast tissue from four female patients (51, 36, 47, and 45 years old). The ovarian cancer MTN contained four pairs of malignant and normal ovarian tissue from four female patients (age 48 – serous papillary cystadenocarcinoma; age 30 – papillary cystadenocarcinoma; age 42 – granulosa-theca cell tumour; age 28 – adenocarcinoma).

Hybridization was performed using 25 ng of a gene-specific ^32^P-labeled DNA probe derived from a Unigene cDNA clone [GenBank accession number W33000]. This gene-specific cDNA fragment was radiolabelled using a Megaprime labelling kit (Amersham Biosciences, Braunschweig, Germany), hybridized overnight at 68°C using ExpressHyb Hybridization Solution (Clontech, Heidelberg, Germany), washed, and exposed to Kodak XAR-5 X-ray film with an intensifying screen (Eastman Kodak Co, Rochester, NY).

### Quantitative RT-PCR

*ERH *mRNA-expression was analyzed with the LightCycler^® ^system (Roche Diagnostics, Germany) in non-tumorigenic and tumorigenic breast cancer cell lines, and formalin-fixed paraffin-embedded breast cancer and normal breast tissue specimens. *GAPDH mRNA *was used as reference to obtain relative expression values. Primers used are presented in Table 2. Real-time RT-PCR was carried out with Fast Start DNA master hybridization probes (Roche Molecular Biochemicals, Germany). PCR conditions were as follows: initial denaturation in one cycle of 15 min at 95°C, followed by 40 cycles at 95°C for 20 sec, 60°C for 20 sec and 72°C for 30 sec. Reaction, data acquisition, and analysis were all done by using the LightCycler^® ^instrument.

**Table 2:** Primers and probes used in real-time RT-PCR

**Gene**	**Primer sequence**	**Product size**
*ERH*	5'-TGAATCCCAACAGTCCCTCT-3'	163 bp
	5'-CGACGAAGGAGCACGTAGAT-3'	
		
*GAPDH*	5'-TGGTCACCAGGGCTGCTT-3'^†^	151 bp
	5'-AGCTTCCCGTTCTCAGCCTT-3'^†^	

### Non-radioisotopic RNA in situ hybridization

Non-radioisotopic RNA ISH was performed as previously described [7,9]. In brief, riboprobes were obtained from plasmids containing cDNA inserts from the same clones used for array hybridization, linearized with restriction enzymes. Probes were digoxigenin-labeled using the Dig RNA labelling kit (Roche Applied Science, Mannheim, Germany). Paraffin embedded tissue specimens were deparaffinized, re-hydrated, washed two times in PBS, and processed according to the manufacturer's instructions (Roche Applied Science, Mannheim, Germany). Hybridized probes were detected using alkaline phosphatase conjugated anti-DIG antibodies and BM Purple as substrate (Roche Applied Science, Mannheim, Germany). After nuclear fast red counter staining (containing 5% aluminium sulphate; VWR International, Dublin, Ireland) sections were examined by a pathologist.

### Statistical analysis

In order to compare the delta CT values of the real time RT-PCR results between specific groups the non-parametric Mann-Whitney-U-test was used.

## Results

### Expression analysis using multiple tissue Northern blots

**Figure 1:**
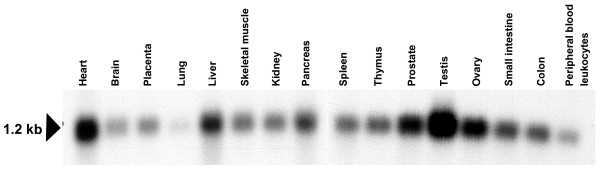
***ERH *expression in human normal tissues analyzed by Northern blot hybridization**. Strong *ERH mRNA *expression was found in normal testis, heart, ovary, prostate and liver. Less abundant *ERH *mRNA was detected in the remaining 11 tissues tested. The human *ERH *transcript is approximately 1.2 kb in size.

*ERH *expression was detected by multiple tissue Northern blots (MTN) in all 16 normal human tissues analyzed (Clontech, Heidelberg, Germany). *ERH *expression was most abundant in testis compared with other normal tissues. Abundant *ERH *expression was also found in normal tissue from the heart, ovary, prostate, and liver. Less abundant expression was detected in the remaining 11 normal tissues, with weakest expression in normal lung tissue. These results are presented in Figure 1.

**Figure 2:**
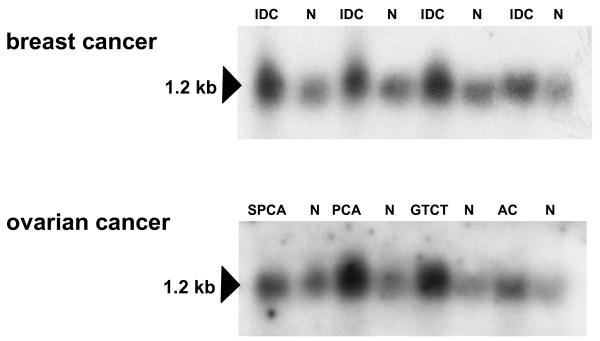
***ERH *expression in breast and ovarian cancer and normal tissue, analyzed by Northern blot**. *ERH *was expressed in all malignant and normal tissue samples. In both tumour entities, *ERH *expression was stronger in all tumour samples as compared with matched and non-matched normal tissue samples. Upper panel: breast cancer; lower panel: ovarian cancer. IDC: invasive ductal carcinoma; SPCA: serous papillary cystadenocarcinoma; PCA: papillary cystadenocarcinoma; GPCT: granulosa-theca cell tumour; AC: adenocarcinoma.

*ERH *expression was further analyzed by MTN in a set of four matched breast cancer/normal breast tissue samples, and a set of four malignant/normal ovarian tissue samples (Clontech, Heidelberg, Germany). *ERH *expression was detectable in all malignant and normal breast tissue samples, and expression was clearly stronger in all tumour samples as compared with normal tissue samples (Figure 2, upper panel). The same expression pattern of more abundant expression in tumour compared with normal tissue samples was also seen in ovarian cancer (Figure 2, lower panel).

### Quantitative RT-PCR

**Figure 3:**
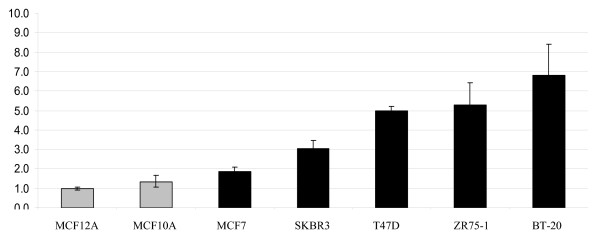
***ERH *expression in benign and malignant breast cell lines. Diagrammatic presentation of quantitative RT-PCR data**. *ERH *expression was analyzed by real-time RT-PCR in the benign human breast cell lines MCF10A and MCF12A (grey columns) and in five breast cancer cell lines (black columns). *ERH *expression, normalized to MCF12A cells (set = 1.0), was significantly more abundant in breast cancer cell lines as compared with benign cell lines (p = 0.05).

In order to investigate the possibility that *ERH *might be differentially expressed in different stages of mammary tumour progression we have compared *ERH *mRNA levels in non-tumorigenic (i.e. MCF10A and MCF12A cells) and five different tumorigenic breast cancer cell lines with LightCycler^® ^RT-PCR (Figure 3). We found that *ERH *expression was significantly more abundant in tumorigenic cell lines as compared with non-tumorigenic cell lines (p = 0.05 according to two-tailed Mann-Whitney U-test). Interestingly, ERH was very abundantly expressed in the highly metastatic breast cancer cell line BT20. These results are presented diagrammatically in Figure 3.

**Figure 4:**
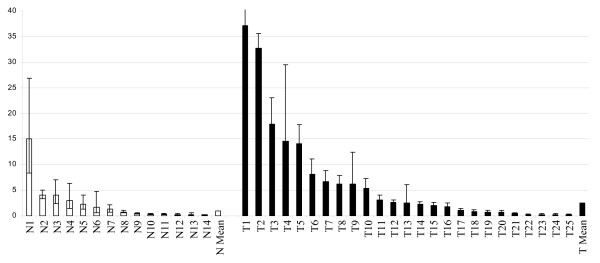
***ERH *expression in normal and malignant breast tissue. Diagrammatic presentation of quantitative RT-PCR data**. *ERH *expression was analyzed by real-time RT-PCR in formalin-fixed paraffin-embedded tissue specimens. Mean *ERH *expression was 2.5-fold more abundant in breast tumours as compared to normal breast tissue (mean expression in normal breast was set = 1.0). N: normal breast; T: invasive breast cancer.

*ERH *expression was further validated by real-time RT-PCR using the LightCycler^® ^system in a set of formalin-fixed paraffin-embedded tissue specimens, consisting of 25 primary invasive breast cancers and 16 normal breast tissue samples. These data are diagrammatically presented in Figure 4. Consistently with the MTN results presented above, mean *ERH *expression in breast tumours was 2.5 fold more abundant than mean *ERH *expression in normal breast tissue (p = 0.1 according to two-tailed Mann-Whitney U-test).

### Cellular localization of ERH mRNA

**Figure 5:**
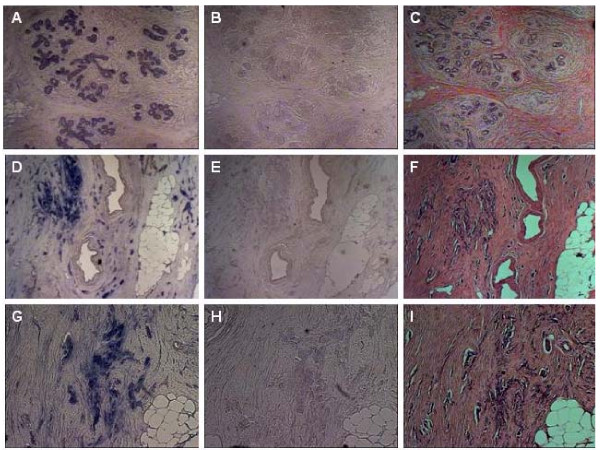
**Representative sections of *ERH *mRNA expression as detected by non-radioactive *in situ *hybridization (ISH)**. A, D, and G: Hybridization with antisense *ERH *probe demonstrates *ERH *mRNA expression. B, E, and H: Hybridization with sense *ERH *probe serves as negative control for the specificity of the antisense probe. C, F, I: Hematoxylin-eosin staining of consecutive sections shown in ISH. *ERH *mRNA was clearly detectable in epithelial cells from invasive breast cancer (D, G), while a less abundant ERH mRNA expression could be detected in epithelial cells of normal breast tissue (A).

Cellular localization of *ERH *mRNA in breast cancer and normal breast tissue was analyzed with non-radioactive RNA ISH. *ERH *expression was up-regulated in breast cancer as compared with normal breast tissue. Representative sections showing *ERH *expression as detected by ISH are presented in Figure 5. *ERH *was specifically and abundantly expressed in the tumour cells of invasive ductal carcinoma (D, G), while a less abundant *ERH *mRNA expression could be detected in the epithelial cells of normal breast lobuli (A).

## Discussion

Thus far, a variety of different functions have been attributed to the ERH protein, including enhancement of pyrimidine biosynthesis, a role in cell cycle regulation, cell growth, repression of transcription and interaction with p21(Cip1/Waf1) [4,12-14]. Moreover, little is known about the role of *ERH *in human malignancies. In the present study, the gene expression pattern of *ERH *has been systematically analyzed in normal human tissues, breast cancer cell lines and a panel of malignant and normal human breast and ovarian tissue samples using three independent methods. Our analysis provides useful insights regarding the still inconsistently defined biological role of *ERH*. It is furthermore the first study to supply a systematic data set concerning *ERH *expression in a human malignancy.

The *Enhancer of rudimentary *gene was first discovered in *Drosophila melanogaster*: Mutations of the *rudimentary gene (r)*, encoding a multifunctional protein for the first three enzymatic activities of the pyrimidine biosynthetic pathway, lead to a characteristic truncation of the wings. Mutation of another gene led to more severely truncated wings in the background of *r-*mutations, and thus this gene was named *Enhancer of the rudimentary *[5]. Later studies provided experimental evidence suggesting that the wild-type ERH protein is a transcriptional (co-)repressor [4,15,16] and its activity is not restricted to the pyrimidine biosynthetic pathway. Another hypothesis was based on the observation that *ERH *is only weakly expressed in non-dividing cell lines of hepatocytes while it is abundantly expressed in fibroblast and hepatoma cell lines, suggesting that *ERH *might have a function necessary for normal cellular proliferation [3].

However, our findings do not support a key role of ERH in cellular proliferation, since *ERH *seems to be heterogeneously expressed in normal tissues expected to be transcriptionally active (more abundant expression in testis, ovary, prostate, and liver, but lower expression in placenta, kidney, pancreas, small intestine and colon – see Figure 1), as well as heterogeneously expressed in terminally differentiated tissues expected to have low transcriptional activity (very weak expression in lung, brain and peripheral blood leucocytes, but abundant in heart and skeletal muscle – see Figure 1).

Based on our finding that *ERH *is abundantly expressed in the majority of normal human tissues analyzed, it does not appear to be an ideal therapeutic drug target in human cancer treatment, but still such a role cannot be completely excluded. On the other hand, more abundant *ERH *expression in tumorigenic as compared with non-tumorigenic breast cancer cell lines (see Figure 3) and the trend of higher expression in malignant as compared with normal tissue samples (see Figure 4) suggest that *ERH *could be possibly used as a prognostic factor in breast cancer. Since similar results were obtained for ovarian cancer (see Figure 2), the expression pattern and the prognostic role of ERH in breast cancer and gynecologic malignancies awaits evaluation in future studies. Furthermore, this expression pattern suggests that *ERH *might be implicated in carcinogenesis and tumour-progression and this should be further investigated in appropriately designed functional studies.

## Conclusion

*ERH *expression is clearly up-regulated in tumorigenic as compared with non-tumorigenic breast cancer cell lines (found by quantitative RT-PCR), and in malignant as compared with normal breast tissue samples (confirmed by three independent methods, i.e. MTN, quantitative RT-PCR, and non-radioisotopic ISH). These findings suggest that *ERH *is progressively up-regulated with tumour progression, and thus it could be used as a prognostic factor in breast cancer. A similar expression pattern was also found in ovarian cancer (by MTN), suggesting that ERH overexpression might be implicated in the initiation and/or progression of other human malignancies as well. Further studies on large breast cancer tissue cohorts are necessary in order to investigate whether ERH could function as a prognostic factor or even a drug target in the treatment of human breast cancer, while functional studies should delineate its possible role in carcinogenesis and tumour-progression.

## Abbreviations

*ERH*: Enhancer of the Rudimentary gene Homologue (*Homo sapiens*); erh: enhancer of the rudimentary gene homologue (species other than *Homo sapiens*); DROER: Drosophila Enhancer of Rudimentary; EST: Expressed Sequence Tag; MTN: Multiple Tissue Northern blot; RT-PCR: Reverse Transcription – Polymerase Chain Reaction; DEPC: Diethylpyrocarbonate; ISH: *In Situ H*ybridization; *GAPDH*: Glyceraldehyde-3-phosphate dehydrogenase; *r*: rudimentary gene (*Drosophila melanogaster*); XERH: Xenopus Enhancer of Rudimentary.

## Competing interests

The authors declare that they have no competing interests.

## Authors' contributions

MZ drafted the manuscript and participated in the design of the study and molecular studies. IL carried out the molecular studies. RK participated in the design and coordination of the study. ED conceived the study, performed the statistical analysis, and participated in its design and coordination, molecular studies, and drafting of the manuscript. All authors have read and approved the final manuscript.

## Pre-publication history

The pre-publication history for this paper can be accessed here:



## References

[B1] Isomura M, Okui K, Fujiwara T, Shin S, Nakamura Y (1996). Cloning and mapping of a novel human cDNA homologous to DROER, the enhancer of the Drosophila melanogaster rudimentary gene. Genomics.

[B2] Jin T, Howard A, Golemis EA, Wang Y, Zhang YZ (2005). Overexpression, purification, crystallization, and preliminary X-ray diffraction studies of the human transcription repressor ERH. Acta Cryst.

[B3] Gelsthorpe M, Pulumati M, McCallum C, Dang-Vu K, Tsubota SI (1997). The putative cell cycle gene, enhancer of rudimentary, encodes a highly conserved protein found in plants and animals. Gene.

[B4] Pogge von Strandmann E, Senkel S, Ryffel GU (2001). ERH (enhancer of rudimentary homologue), a conserved factor identical between frog and human, is a transcriptional repressor. Biol Chem.

[B5] Wojcik E, Murphy AM, Fares H, Dang-Vu K, Tsubota SI (1994). Enhancer of rudimentaryp1, e(r)p1, a highly conserved enhancer of the rudimentary gene. Genetics.

[B6] Schmitt AO, Specht T, Beckmann G, Dahl E, Pilarsky CP, Hinzmann B, Rosenthal A (1999). Exhaustive mining of EST libraries for genes differentially expressed in normal and tumour tissues. Nucl Acids Res.

[B7] Dahl E, Sadr-Nabavi A, Klopocki E, Betz B, Grube S, Kreutzfeld R, Himmelfarb M, An HX, Gelling S, Klaman I, Hinzmann B, Kristiansen G, Grützmann R, Kuner R, Petschke B, Rhiem K, Wiechen K, Sers C, Wiestler O, Schneider A, Höfler H, Nährig J, Dietel M, Schäfer R, Rosenthal A, Schmutzler R, Dürst M, Meindl A, Niederacher D (2005). Systematic identification and molecular characterization of genes differentially expressed in breast and ovarian cancer. J Pathol.

[B8] Klopocki E, Kristiansen G, Wild PJ, Klaman I, Castanos-Velez E, Singer G, Stohr R, Simon R, Sauter G, Leibiger H, Essers L, Weber B, Hermann K, Rosenthal A, Hartmann A, Dahl E (2004). Loss of SFRP1 is associated with breast cancer progression and poor prognosis in early stage tumours. Int J Oncol.

[B9] Zafrakas M, Chorovicer M, Klaman I, Kristiansen G, Wild PJ, Heindrichs U, Knüchel-Clarke R, Dahl E (2006). Systematic characterization of *GABRP *expression in sporadic breast cancer and normal breast tissue. Int J Cancer.

[B10] Veeck J, Chorovicer M, Naami A, Breuer E, Zafrakas M, Bektas M, Dürst M, Kristiansen G, Wild PJ, Hartmann A, Knuechel R, Dahl E (2008). The extracellular matrix protein ITIH5 is a novel prognostic marker in invasive node-negative breast cancer and its aberrant expression is caused by promoter hypermethylation. Oncogene.

[B11] Koensgen D, Mustea A, Klaman I, Sun PM, Zafrakas M, Lichtenegger W, Denkert C, Dahl E, Sehouli J (2007). Expression analysis and RNA-localization of *PAI-RBP1 *(*SERBP1*) in epithelial ovarian cancer: association with tumour progression. Gynecol Oncol.

[B12] Wan C, Tempel W, Liu ZJ, Wang BC, Rose RB (2005). Structure of the conserved transcriptional repressor enhancer of rudimentary homolog. Biochemistry.

[B13] Smyk A, Szuminska M, Uniewicz KA, Graves LM, Kozlowski P (2006). Human enhancer of rudimentary is a molecular partner of PDIP46/SKAR, a protein interacting with DNA polymerase delta and S6K1 and regulating cell growth. FEBS J.

[B14] Lukasik A, Uniewicz KA, Kulis M, Kozlowski P (2008). Ciz1, a p21 cip1/Waf1-interacting zinc finger protein and DNA replication factor, is a novel molecular partner for human enhancer of rudimentary homolog. FEBS J.

[B15] Kwak YT, Guo J, Prajapati S, Park KJ, Surabhi RM, Miller B, Gehrig P, Gaynor RB (2003). Methylation of SPT5 regulates its interaction with RNA polymerase II and transcriptional elongation properties. Mol Cell.

[B16] Amente S, Napolitano G, Licciardo P, Monti M, Pucci P, Lania L, Majello B (2005). Identification of proteins interacting with the RNAPII FCP1 phosphatase: FCP1 forms a complex with arginine methyltransferase PRMT5 and it is a substrate for PRMT5-mediated methylation. FEBS Lett.

